# Pathway-centric visualization of cell-cell communication in single-cell transcriptomics data

**DOI:** 10.1038/s41540-026-00768-2

**Published:** 2026-06-20

**Authors:** Sidrah Maryam, Martin Manolov, Rafael Kramann, Sikander Hayat

**Affiliations:** 1 Department of Medicine 2, Uniklinik, Aachen, NRW Germany; 2 Institute for Computational Genomics, Uniklinik, Aachen, NRW Germany; 3 Cardiovascular Research Institute and Department of Medicine, Icahn School of Medicine at Mount Sinai, New York, NY USA; 4 Windreich Department of Artificial Intelligence and Human Health, Icahn School of Medicine at Mount Sinai, New York, NY USA

**Keywords:** Cell biology, Computational biology and bioinformatics, Systems biology

## Abstract

Single-nuclei transcriptomics enables investigating ligand-receptor mediated cell-cell communication between different cell-types. However, current tools do not allow for non-programmatic means to access this analysis. Additionally, most methods can not account for molecular pathways involved in downstream receptor signaling in cell-cell communication. We developed scVizComm, a ShinyApp based web-portal that can be used to interactively visualize ligand-receptor networks across cell-types and different conditions. scVizComm can be used to host pathway centric pre-calculated cell-cell communication results. Using human kidney and heart single-nuclei transcriptomics data, we showcase the utility of scVizComm in understanding ligand-receptor interactions involved in fibrosis related pathways. Additionally using mouse anterior brain data, we demonstrate the major ligand-receptor expression exploration in space. scVizComm allows interactive visualization and pathway centric prioritization of cell-cell communication analyses.

## Introduction

Computational tools to analyze cell-cell communication (CCC) have become a mainstay of typical single-nuclei transcriptomics (snRNA) analyses. CCC is pivotal to understanding cellular crosstalk that governs cellular behavior in homeostasis, differentiation and disease progression. Multiple computational tools have been developed to identify putative ligand-receptor interactions that mediate CCC across cell-types^1^. Moreover, network topology based methods have also been developed to identify condition-specific ligand-receptor interactions^2^. Recently, a few tools have also been implemented to infer downstream activity of potential ligand-receptor interactions^3^. First generation of CCC methods provided a ranked list of condition agnostic ligand-receptors, while methods like CrossTalkeR spearheaded the comparative analyses across conditions^4^. Some methods can also compare CCC across samples for exploration of LR interactions^5^. However, all these methods require programmatic handling which severely limits the use of these tools for non-coders. Additionally, most tools do not account for underlying well-studied genesets and pathways that might play a role in downstream activity due to the putative ligand-receptor interactions.

To overcome these limitations, we have created scVizComm, an interactive visualization tool to display pathway and associated ligand-receptor interactions. This shiny app implementation of the tool would allow users to explore deeper insights of the cellular interactions obtained from fundamental CCC methods. scViZComm has five unique features (1) Visualize condition-wise Ligand-Receptor interaction for the source and target clusters of choice, (2) Determine expression dependent LR Score, (3) Determing the geneset expression distribution in the data associated with the selected pathway using AUCell^6^ (4) Downstream pathway association of aggregated receptors per condition or cluster KEGG pathway^7^ analysis, and (5) Spatial data visualization of selected ligand-receptor present in the data. scVizComm can be accessed online through our shinyApp.

## Results

We performed scVizzComm assisted single cell data analysis on three dataset—the human heart^8^ and kidney^9^ datasets, interferon-beta stimulated human Peripheral Blood Mononuclear cells Seurat data^10^. We also used spatial data analysis on seurat demo brain dataset^11^ to showcase the utility of scVizzComm in showing selected LR pairs on space using 10x visium data:

### Use case 1: Interactive visualization of ligand-receptors and downstream pathways in Myocardial Infarction (MI) Human data

**Fig. 1:**
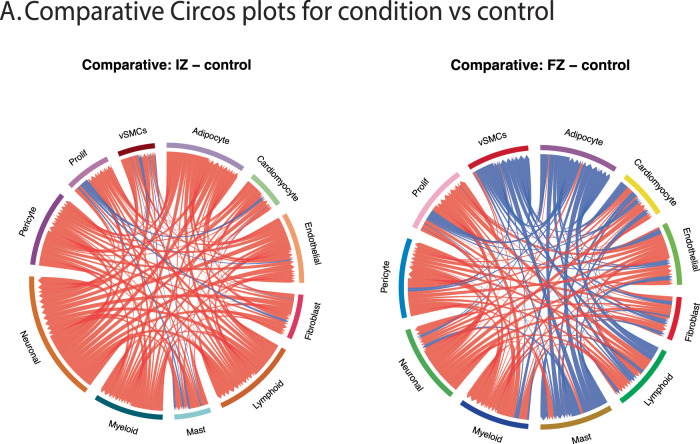
Implementation of scVizzComm on Myocardial Infarction data. Differential Circos plots depict the increased or lost interactions in different conditions between different cell types in MI dataset.

Here, we used myocardial infarction data of human heart from left ventricle containing 191,795 cells to demonstrate the application of scVizzComm in visually exploring ligand-receptors and downstream pathways. Briefly, the data consists of four different conditions including control, tissue from the Fibrotic zone (FZ), myocardial infarction ischemic zone (IZ), and relatively unaffected area of myocardial infarction (Remote zone - RZ)^8^. We focused on significant ligand-receptor interactions obtained from cellphonedb, with *p*-value less than 0.05. In differential comparison analyses, we found overall decreased interactions in the fibrotic and ischemic zone (Fig. 1). For downstream workflow analyses, we focused on Cardiomyocytes interaction with mast cells in fibrotic zone with respect to control, to study the changes involving cell-cell communication between the immune and cardiac cells.

To showcase the ligand-receptor-pathway relationship, we selected NCAM1 as a representative ligand in FZ vs control comparison. NCAM1 (Neural Cell Adhesion Molecule 1) is a well known cell surface glycoprotein essential for mediating cellular and cell-matrix adhesion^12^. Additionally, NCAM1 is known to interact with multiple receptors and downstream effectors, positioning it as an ideal candidate to dissect potential pathway crosstalk in FZ condition. Increased expression of NCAM1 receptors were noted in the fibrotic zone, thereby suggesting the enhanced signaling in cardiomyopathy^13^ (Supplementary Data 1–2).

**Fig. 2:**
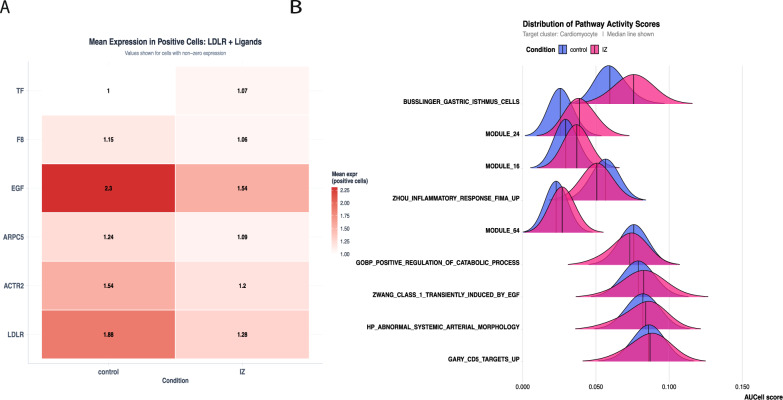
Implementation of scVizzComm on Myocardial Infarction data. **A** Mean expression of selected receptor of users choice and its ligands. **B** Ridge plot showing the distribution of genes associated with pathway in which the selected receptor exists.

**Fig. 3:**
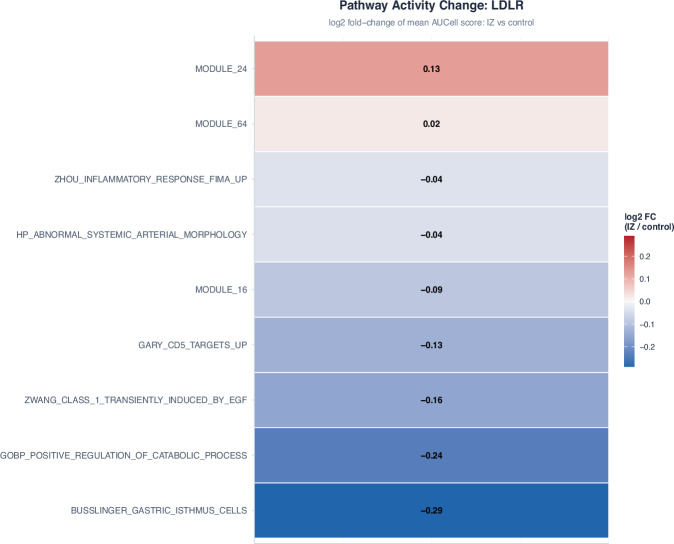
Implementation of scVizzComm on Myocardial Infarction data. Mean pathway differential score between conditions for the pathways associated with receptor.

Further, we looked for Receptor-associated pathway downstream information in the dataset. In this panel, we chose LDLR receptor associated with multiple heart conditions^14^. The expression of associated ligands were presented in control and IZ (Fig. 2A). Besides, the distribution of gene sets associated with pathways in which receptor LDLR exists, was plotted as ridge plot comparing the two conditions, and mean score of those pathway was shown as heatmap (Figs. 2B–3).

**Fig. 4:**
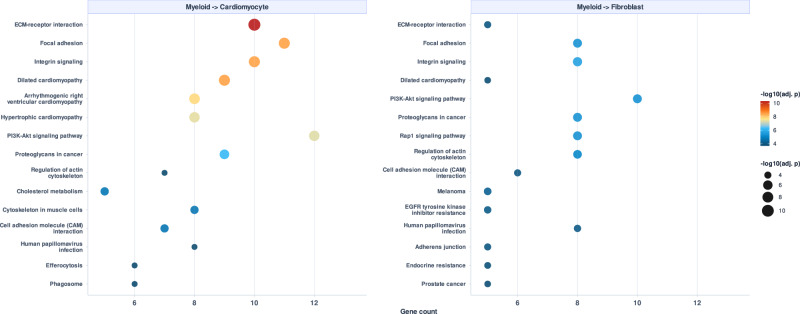
Implementation of scVizzComm on Myocardial Infarction data. KEGG analysis dotplot shows the different pathway regulation of receptors found in cardiomyocytes and fibroblasts in response to interaction with myeloid cells.

To further determine the downstream co-functioning of different receptors in target cluster, KEGG pathway analysis is performed for the target cluster of interest. Here, the analysis can be done using two different combinations. In the comparative case, the source and target cluster are chosen, and depending on the differences between the receptor activity, the enrichKEGG is performed, showing how the same clusters cause different pathway regulation in different conditions. Second way of exploring the interactions provided by this panel involves the same experimental condition comparison. In this case, after choosing a condition and source cluster, one or more target clusters are selected showing the pathway signaling from same source cluster causes different pathway activity in different target clusters. Here, we showed an example of the pathway enrichment of receptors from cardiomyocytes and fibroblasts triggered by the ligands from myeloid (Fig. 4). We focused on cardiomyocyte, fibroblast and myeloid interaction to showcase the KEGG activity in IZ condition.

To note the differences in ligand-receptor pair in different conditions, with same set of target and source cluster, scatter plot is generated in the next tab. Here, we see the ligand-receptor pairs that are common in control and FZ are spread on the quadrant, however, the ones that are unique to the condition can be seen on the x or y axis respectively(Supplementary Data 3). This interactive plot shows us the interactively the LR pairs with their scores in respective condition. We see NCAM1-CACNA1C to be strongly interacting LR pair in FZ as compared to control^15^.

### Use case 2: scVizzComm based analyses of the Kidney Precision Medicine Project (KPMP) data

**Fig. 5:**
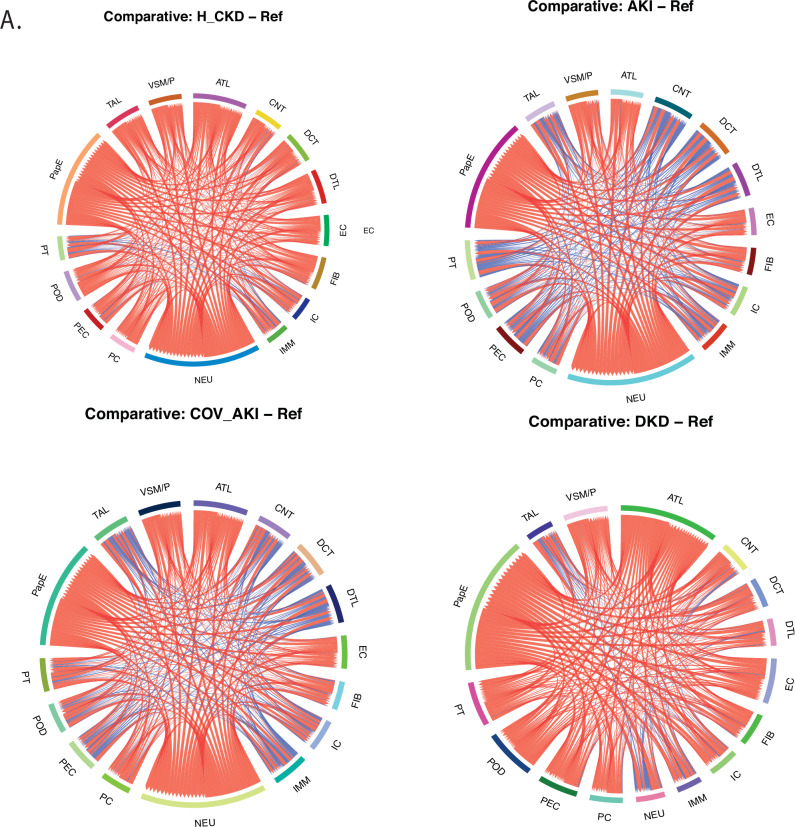
Comparative analyses of the human kidney KPMP dataset interactions. Differential Circos plots depict the increased or lost interactions in different conditions between different cell types in KPMP human kidney dataset.

The kidney dataset contain 200338 cells from Kidney Precision Medicine Project(KPMP)^9^, consisting of healthy kidney tissue and several kidney diseases including chronic kidney disease(CKD), hypertension associated CKD (H_CKD), diabetic kidney disease (DKD), acute kidney injury (AKI), and covid induced AKI (COV_AKI). The LR interactions were calculated using LR scores obtained from preprocessing. The differential patterns of cellular interactions observed in each pathological condition relative to the reference was found to be largely diminished (Fig. 5). To further investigate this phenomenon, we specifically examined the interactions between immune cells and proximal tubule (PT) cells, given their potential role in modulating local tissue responses. We focused mostly on H_CKD with respect to control.

Given the pivotal role of the EGFR receptor in regulating cell proliferation, survival, and fibrotic remodeling, and its previously reported involvement in kidney disease progression^16^, we selected EGFR as a representative node for to determine the relationship between ligand-recetpor-pathway interaction. Notably, EGFR exhibited increased interaction density in the kidney injury condition (Supplementary Data 4–5), underscoring its potential contribution to pathological signaling within the diseased microenvironment.

**Fig. 6:**
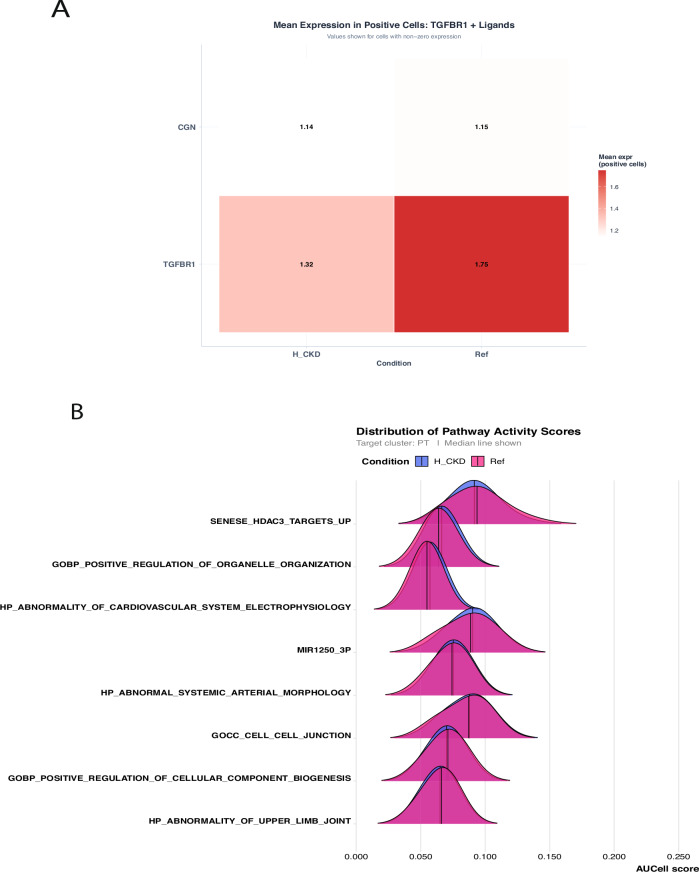
Implementation of scVizzComm on KPMP data. **A** Mean expression of selected receptor TGFRB1 and its ligands in different condition. **B** Ridge plot showing the distribution of genes associated with pathway in which the receptor exists.

To look for the pathway downstream expression of TGFRB1 with its ligands was focused to compare between H_CKD and reference(Fig. 6A). Further, the AUCell score of the pathways associated with the receptor were depicted as ridge plot and mean scores as the heatmap (Fig. 6B, Fig. 8).

**Fig. 7:**
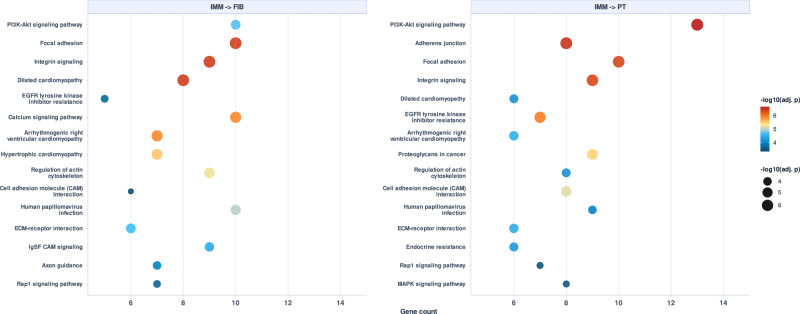
Comparative analyses of the human kidney KPMP dataset interactions. KEGG analysis performed on receptors present in fibroblast and PT cells that interact with Immune cells in H_CKD.

To compare between the pathway activity of different conditions for same condition and ligand cluster, for different target cluster, KEGG analysis was performed for receptors in PT and Fibroblast cells that interact with ligands of Immune (Fig. 7).

Next, we focused on the unique pairs of ligand-receptor present in condition vs reference, and we identified the HSP8-LRP2 pair as uniquely present and highly expressed in the H_CKD condition, specifically within immune and proximal tubule cell interactions. The receptor has been previously implicated in mediating kidney injury and disease progression^17^ (Supplementary Data 6).

### Use case 3: Interferon-beta1(Ifnb) Peripheral Blood Mononuclear Cells (PBMC) dataset

Here, we focused on Interferon-beta1(Ifnb) Peripheral Blood Mononuclear Cells (PBMC) dataset, that contains 13,999 cells across 14,053 features^10^. In this experiment, the PBMCs were split into control group and stimulated group, where the stimulated group was treated with interferon beta. The data comprises 13 cell types, where we focus on CD14(cluster of differentiation) and natural killer (NK) cells. We implemented liana(0.1.13) pipeline to calculate cell-interactions, and AUCell(1.26.0) to calculate pathway activity for each cell. Then, the CCC exploration was performed^6^. We found enhanced interaction between cell types in stim condition vs control (Fig. 9A). For ligand-receptor-pathway relationship we looked for CXCR4 in in NK cells(Supplementary Data 7). Further, we saw unique expression of CXCR3 in stim(Supplementary Data 8)^18^. We focused on cell types CD14 mono cells interacting with NK cells, and found enhanced KEGG pathway activity in stim condition (Fig. 10). We further explored the downstream analysis of receptor CXCR4, in NK cells, associated with cell migration and development^19^. The expression of receptor and ligand was found to be more in the control (Fig. 11A). The pathway score distribution (Fig. 9B) and mean pathway differential score was depicted pathways for CXCR4 (Fig. 11B).

**Fig. 8:**
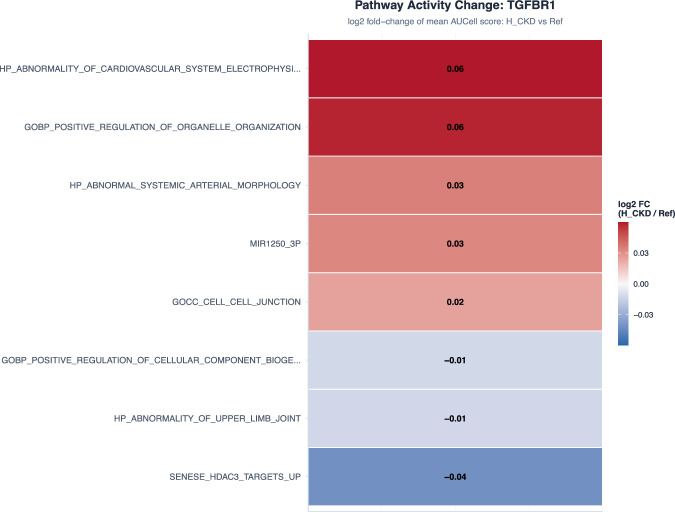
Implementation of scVizzComm on KPMP data. Mean pathway score change activity for the pathways associated with receptor in condition with respect to control.

**Fig. 9:**
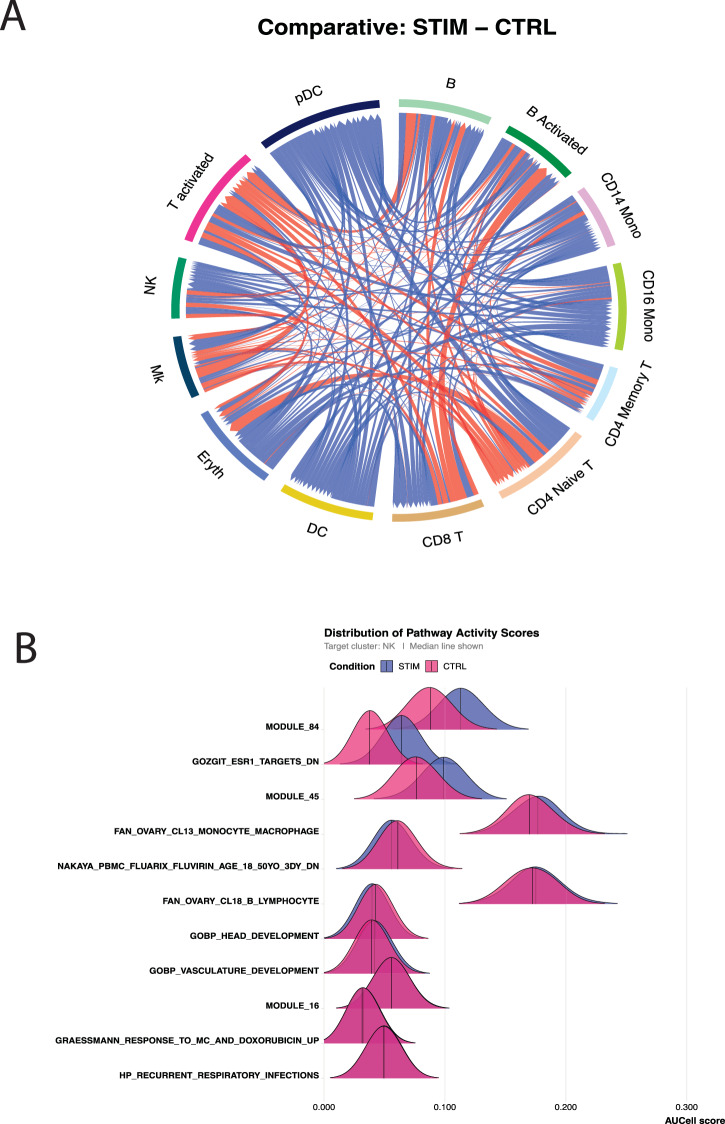
Implementation of scVizzComm on IFNB Seurat data. **A** Circos plots show the differential interaction between different immune cells in stim condition vs control. **B** Ridge plot show the distribution of AUCell pathway score associated with CXCR4.

**Fig. 10:**
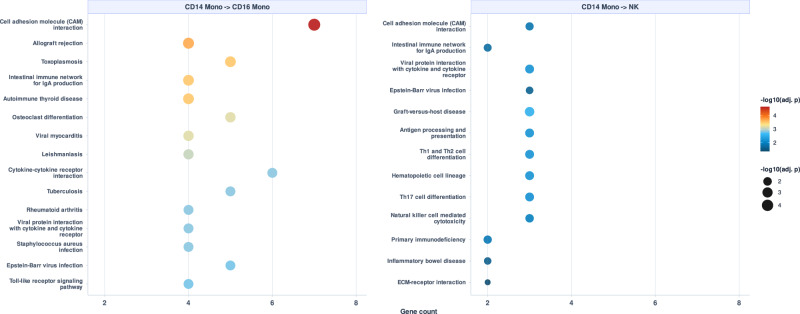
Implementation of scVizzComm on IFNB seurat data. KEGG analysis show increase in the pathways related to disease in stim condition.

**Fig. 11:**
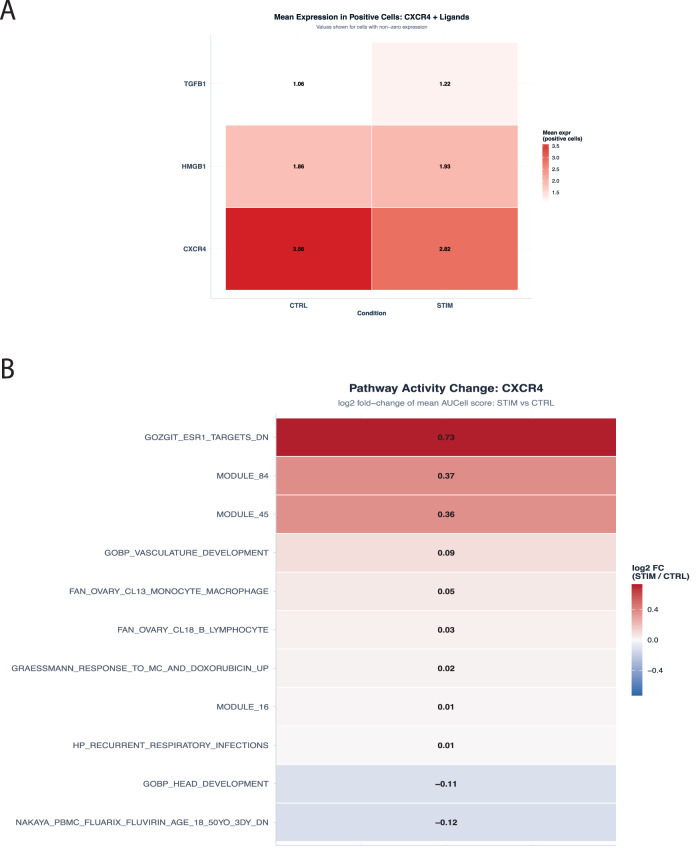
Implementation of scVizzComm on IFNB seurat data. **A** Mean expression of selected receptor CXCR4 and its ligands in different condition. **B** Mean pathway score change activity for the pathways associated with receptor in different conditions.

### Use case 4: Spatial data analysis

**Fig. 12:**
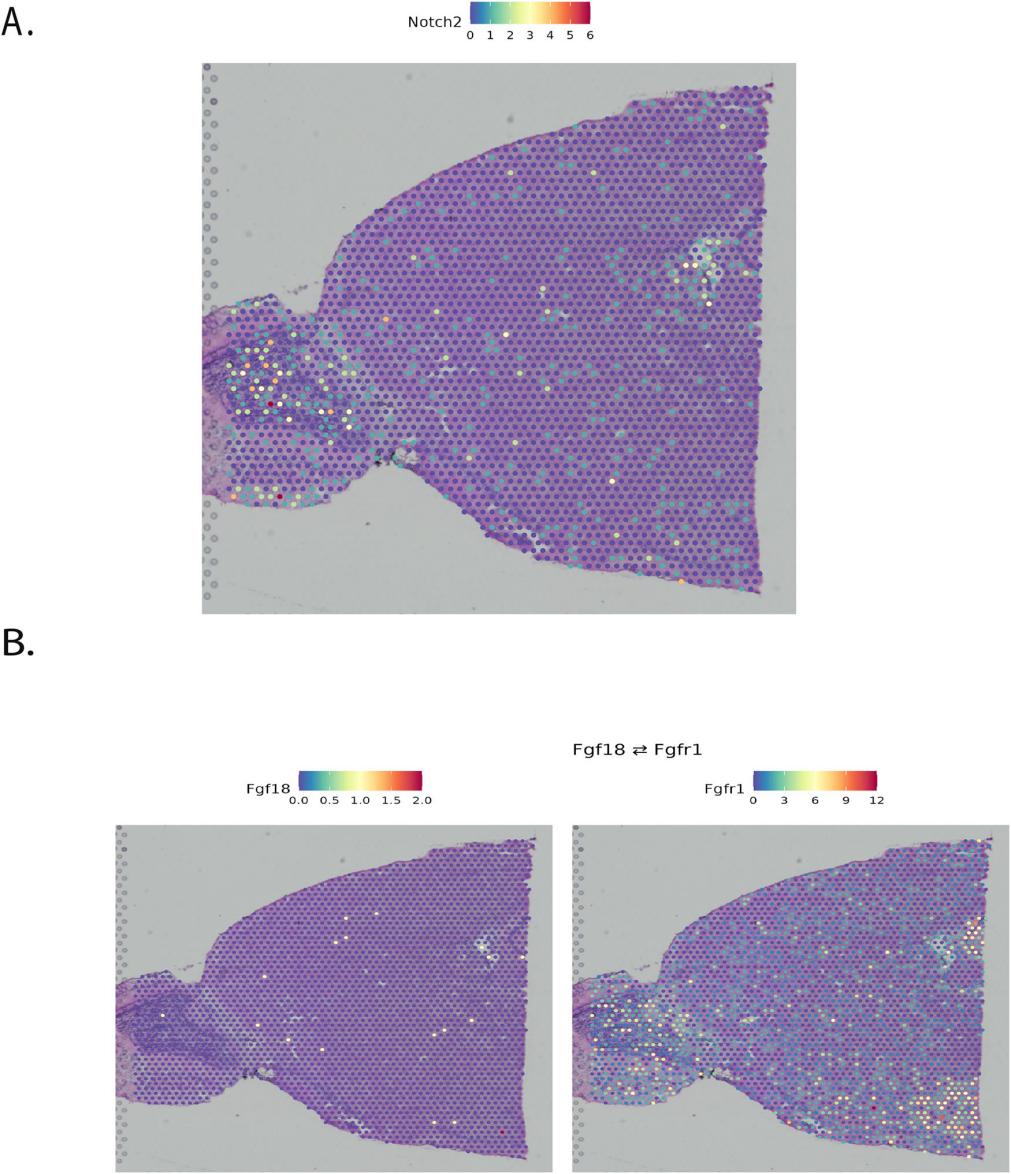
Implementation of scVizzComm on spatial data. **A** Expression of gene of choice on spatial tissue. **B** Expression and spread of ligand-receptor, found in cellphonedb database, on tissue.

To explore the spatial data, the standard seurat brain dataset from mouse was utilized^20^. The dataset is the sagital mouse brain slices generated by visium v1 chemistry. The data contains two sections - anterior and posterior. However, we loaded the anterior section of the brain data. Here, the structure of seurat object contains spot by gene expression matrix, H*&*E image from the tissue. For demonstration, we used Notch2 gene associated with the brain development in mouse^21^ (Fig. 12A), and found its expression quite high in the hippocampus region.

To investigate a pair of ligand-receptor shown on the spatial slide of mouse brain, Fgf18-Fgfr1 pair was used. This LR pair is associated with differentiation in mouse skull and hence, development of brain^22^. It can be noted that the expression of ligand Fgf18 is scattered in the mid-brain region, while the fgfr1 receptor is expressed highly in the tissue^23^ (Fig. 12B).

## Discussion

snRNA-Seq allows us to address cell-cell communication which is mediated by ligand-receptor(LR) interactions and influences downstream signaling and pathways. While several computational tools exist for LR analysis, they often lack accessibility for non-programming users and typically do not incorporate pathway-level interpretation of downstream receptor signaling for receptors of choice. To address these limitations, we developed scVizComm, a user-friendly ShinyApp that enables interactive exploration of pre-computed LR networks across different cell types and biological conditions. One of the key innovations of scVizComm is its focus on pathway-centric analysis, allowing users to move beyond lists of LR pairs and towards understanding their functional roles within broader signaling cascades. Applying scVizComm to human kidney, heart and PBMC snRNA-seq datasets, we demonstrate its utility in highlighting fibrosis-associated pathways, including TGF-*β*, Notch, and integrin signaling. This pathway-centric approach allows researchers to prioritize biologically meaningful LR interactions, particularly those converging on shared fibrotic mechanisms. Additionally, scVizComm facilitates hypothesis generation by providing an intuitive interface for exploring differential LR activity across conditions, such as healthy versus diseased tissue. Despite its strengths, scVizComm currently relies on LR inference results, meaning upstream data processing and statistical modeling must be performed externally. For spatial data, scVizComm provides exploring the expression of genes, ligand-receptor on the tissue slide and hence, showing the region of dense expression. Future development could integrate these pipelines directly into the platform, allowing end-to-end analysis within a single interface. Nonetheless, scVizComm represents a significant step forward in making LR network analysis more accessible, interpretable, and functionally relevant. Its pathway-centric visualization paradigm offers a scalable and flexible solution for researchers seeking to uncover the mechanistic underpinnings of cell-cell communication in health and disease.

scVizzComm is a user-friendly shiny application that allows users to determine the downstream signaling of cellular interactions. It provides an accessible and interactive platform for visualizing ligand-receptor networks with a novel focus on pathway-centric interpretation. By enabling users to explore cell-cell communication in the context of biologically relevant signaling pathways, scVizComm bridges a critical gap between complex transcriptomic data and functional insight. Its user-friendly interface empowers researchers from diverse backgrounds to generate and prioritize hypotheses, particularly in disease contexts such as fibrosis. While future integration of upstream analytical pipelines will further enhance its utility, scVizComm serves as a powerful tool for advancing mechanistic understanding of intercellular signaling in complex tissues. Importantly, the platform democratizes access to LR analyses, supporting scientists without extensive computational backgrounds and enabling collaborative interpretation in translational research settings.

## Methods

scVizComm relies cell interaction output obtained from any already available cell interaction tools. The user provides 3 files separately for full exploration on the application. First, the cell interaction file in rds or csv format, consisting of mandatory columns ligand, receptor, src_cell_type, tgt_cell_type, condition, lr_score. Second file, the seurat object with UMAP for overview of dataset, containing cell type in column cell_type_original, and different groups in ’treatment’ and assay name ’originalexp’. Third file must contain the pathway scores per cell using any pathway activity method in rds or csv format, and consist of columns cell_id, cell_type, condition, pathway_name, score. The user can provide one or all of the files as per need. The panels dependent on the uploaded file will be enabled as it the data is loaded.

**Fig. 13:**
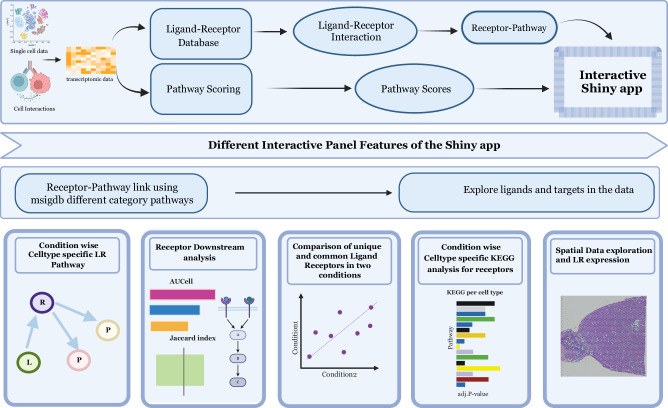
The schematic representation of the scVizzComm workflow. The preprocessing step is implemented on clustered and annotated data as a seurat object. Further, the ligand-receptor information is calculated using publicly available ligand-receptor database, and pathway scoring tool to determine pathway activity per cell. After uploading the dataset in the application, the ligand-receptor-pathway relationship is determined using msigdb. The output files from these steps are used as input to scVizzComm for further exploration of cell-cell communication across conditions and cell-types.

The entire shiny app is written in R language to explore CCC output results (Fig. 13).

The first step involves computational processing of the data.

The pre-processing of the data can be performed using any method framework, and the resulting output files serve as the input for shiny app. The files should contain the important column names, as the downstream processing depends on it. The application offers a range of interactive, customizable features to facilitate in-depth exploration and interpretation of the cell-cell communication landscape derived from the analysis outputs.

### Overview

scVizzComm can be utilized to schematically conduct basic data exploration on a 2D UMAP embedding to display data in each cluster and condition. Additionally, from a list of genes available in the dataset, the gene expression of interest can be obtained as a heatmap in different clusters and condition.

### Circos plots

Based on the LR Score calculated from CCC output from any conventional method, the overall and comparative interaction between different cell types and conditions is visualized. The edge thickness corresponds to the number of ligand-receptor interactions and the weight determines the LR Score. On selecting comparative circos plots, the plot representing the differential LR Scores for interactions are plotted between the conditions for all cell types.

The differential LR Scores are calculated using:

1 $$\,{\rm{LRdiff}}={{\rm{LRScore}}}_{{\rm{cond1}}}-{{\rm{LRScore}}}_{{\rm{cond2}}}$$

Where, LRScore_cond1_ and LRScore_cond2_ represent the LR scores for *condition1* and *condition2*, respectively. The LRdiff quantifies the extent to which *condition1* is different relative to *condition2*. The positive interactions are shown in green color, and negative are shown in red.

### Ligand-receptor relationship

In order to investigate the ligand-receptor-pathway (LRP) relationship, one of the respective nodes (e.g., ligand-receptor) needs to be chosen by the user. Once a node type is selected, the corresponding ligand/receptor are presented on the drop down list to choose. On choosing a node, the sankey plot is generated showing the relationshiop between the ligand-receptor-pathway. The user can choose the category of pathway to show the pathway relationship. Here, msigdb database(7.5.1) is used to find the pathway-receptor association. A sankey graph with edges pointing from ligands to receptors and pathway associated with the receptor is listed using the sankeyNetwork function from the networkD3 package (3.0.4.1). This provides the relatedness between ligand and receptor, and the pathways in which the receptor occur. The expression of ligand and receptor in the dataset is also calculated.

### Receptor-associated pathway downstream

The aim of this panel is to visualize the pathway level activity of a selected receptor in the target cluster. Heatmap is generated depicting the mean expression of associated ligands in the two conditions. Further, a table is generated that shows the composition of cells expressing the ligand or receptor in each condition. The msigdb pathways associated with the chosen receptor are listed, and their AUCell score is presented as a heatmap comparison between the two conditions, as well as, the distribution of AUCell score for the pathways are shown in each condition as a ridge plot.

### KEGG pathway analysis in target cluster

This panel of the application focuses on the KEGG pathway analysis on the aggregated set of receptors in user-defined target cluster. The user is provided with two options of (a) selecting across or (b) same condition analysis to determine the pathway up-regulation dependent on receptors. In this panel, a source and target cluster is selected. Based on the receptors present in the target cluster, enrichKEGG function is used to perform KEGG analysis using clusterprofiler(4.12.6)^7^. This gives us an overview of how the different pathways are up-regulated in different conditions with similar pattern of source and target clusters. On using constant condition, the differences in pathway regulation for different target clusters with same source cluster can be determined.

### Evaluation of differential Ligand-Receptor networks across conditions

This panel is useful for determining common and uniquely expressed LR pairs in two conditions. Here, the user provides the conditions, source, and target clusters of interest, to establish the LR pairs and the strength of interaction(LR Score). The scatter plot is produced where uniquely interacting pairs for condition1 are shown on the x and for condition2 are shown on y axis, while the common pairs are scattered based on the LR Score in the quadrant.

### Spatial expression

This panel is dedicated to the visualization of visium 10x/spatial transcriptomics data. Here, once the data is loaded, all the genes present in the data are provided as the choice to determine their expression in space. On selecting the gene of interest, the gene is plotted on the entire slide. This depicts the regions of high density of expression for the gene. For the CCC exploration, the entire data set is searched for the expressed ligand-receptor pairs present in the underlying cellphonedb database^24^. All such pairs existing in the data are listed. From the list, the pair of interest can be chosen that provides the expression of ligand-receptor on slide, thereby, showing the regions of high and low expression.

The stepwise usage of the application is described in Supplementary Figs. 1–7. The input file is provided to the application, where for each panel, the respective conditions and clusters are selected for in-depth interaction between groups. Further, the results provide the interaction informations as pictorial plots, and generated tables.

## Supplementary information

Supplementary information

Supplementary data

## Data Availability

Data Availability: The scVizzComm application is deployed on https://costalab.ukaachen.de/shiny/smaryam/ with the preloaded data of heart, kidney and spatial brain dataset. The Figures and Supplementary Data are uploaded at 10.5281/zenodo.15363559.Code Availability: The code for preprocessing and application is available at https://github.com/hayatlab/scVizComm.

## Code availability

The code for preprocessing and application is available at https://github.com/hayatlab/scVizComm.
