# Supplementary material for: Pathway-centric visualization of cell-cell communication in single-cell transcriptomics data
Source: NPJ Syst Biol Appl. 2026 Jun 20;12:89. doi: 10.1038/s41540-026-00768-2 (PMC13283212; doi:10.1038/s41540-026-00768-2)
Supplement: Supplementary file 1 — Supplementary information [file 41540_2026_768_MOESM1_ESM.pdf]

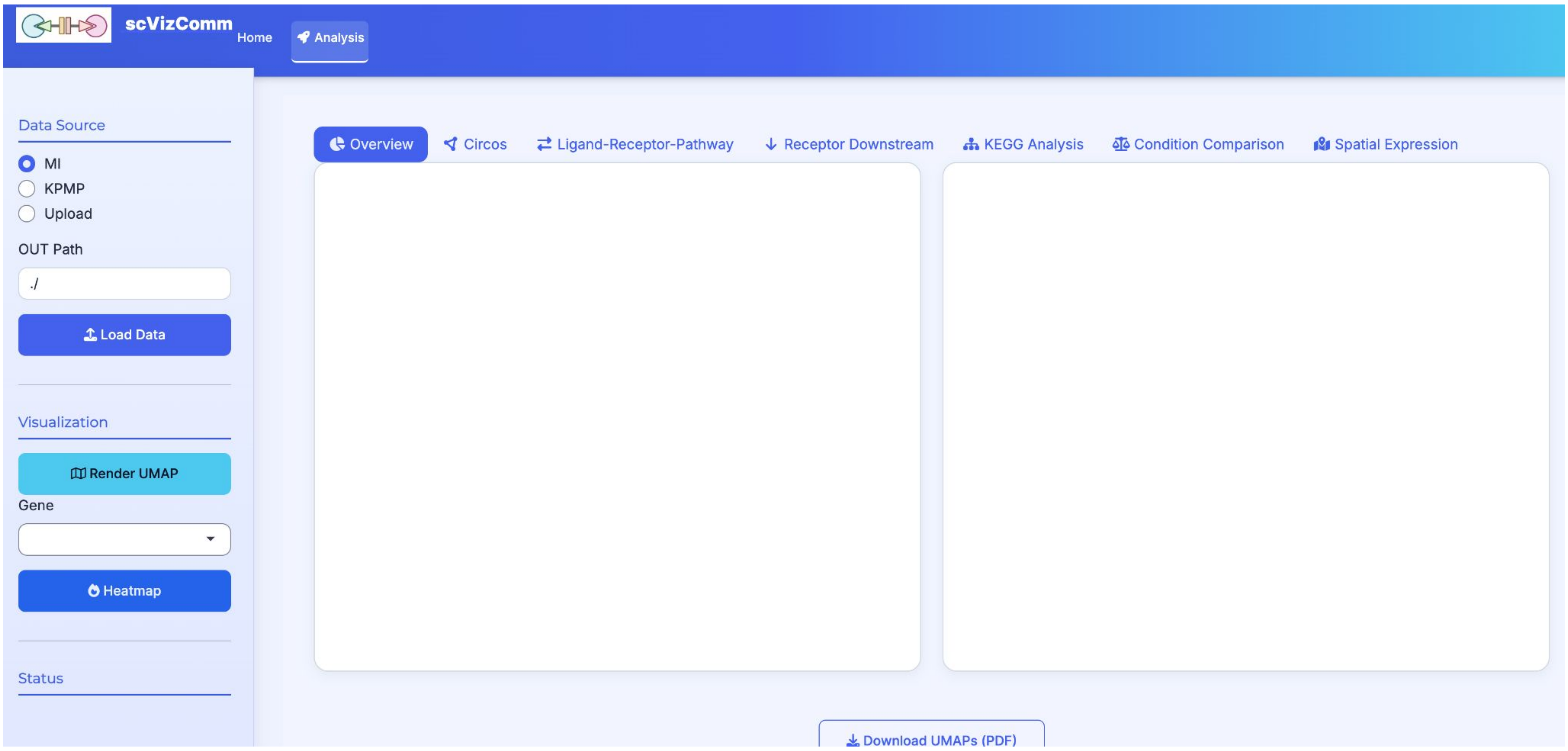

Supplementary Figure 1:
Presentation of different panels present in the application.

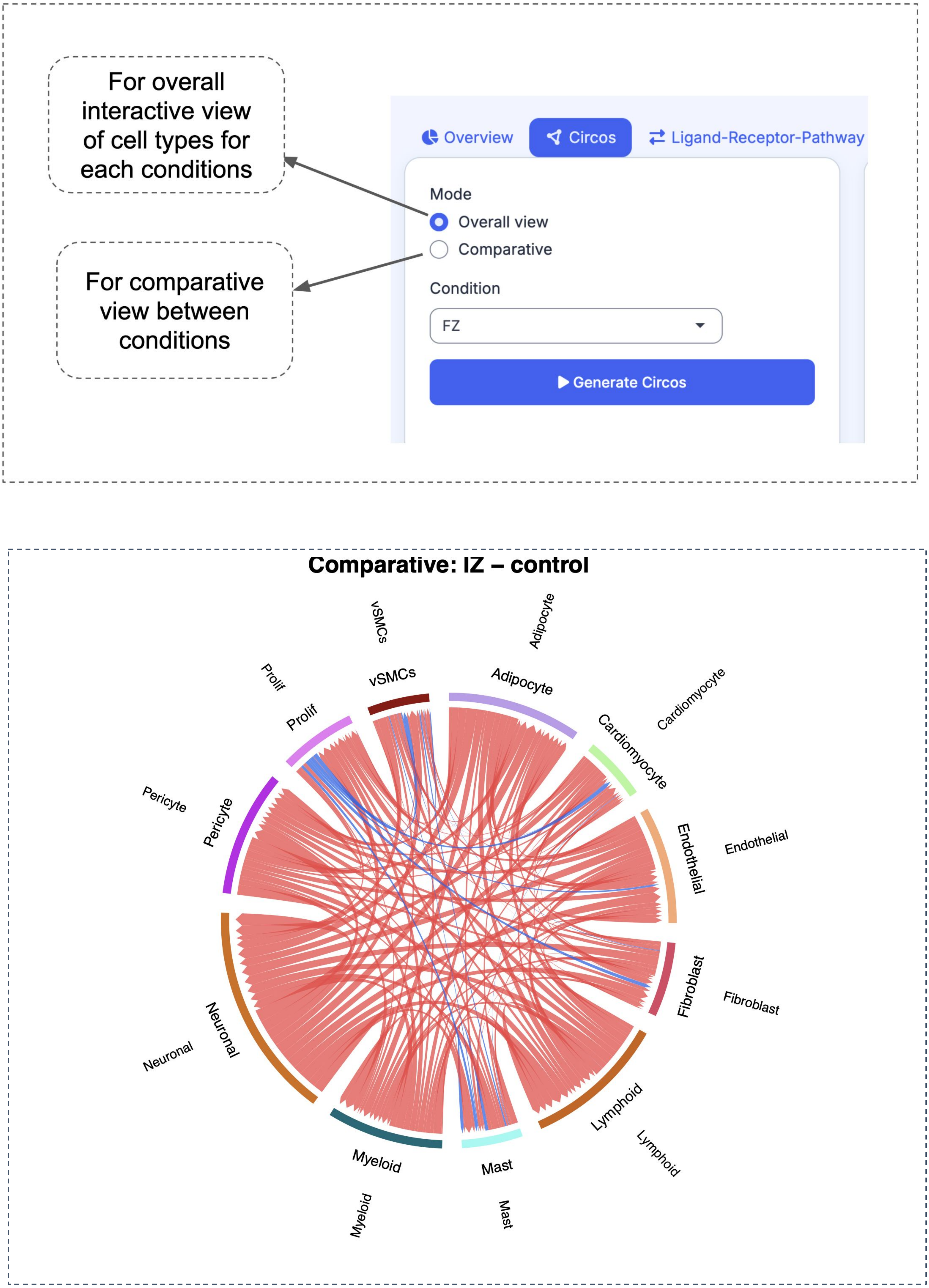

Supplementary Figure 2 :
Circos plot showing the different interaction between cell types for each condition, or difference of interaction between condition.

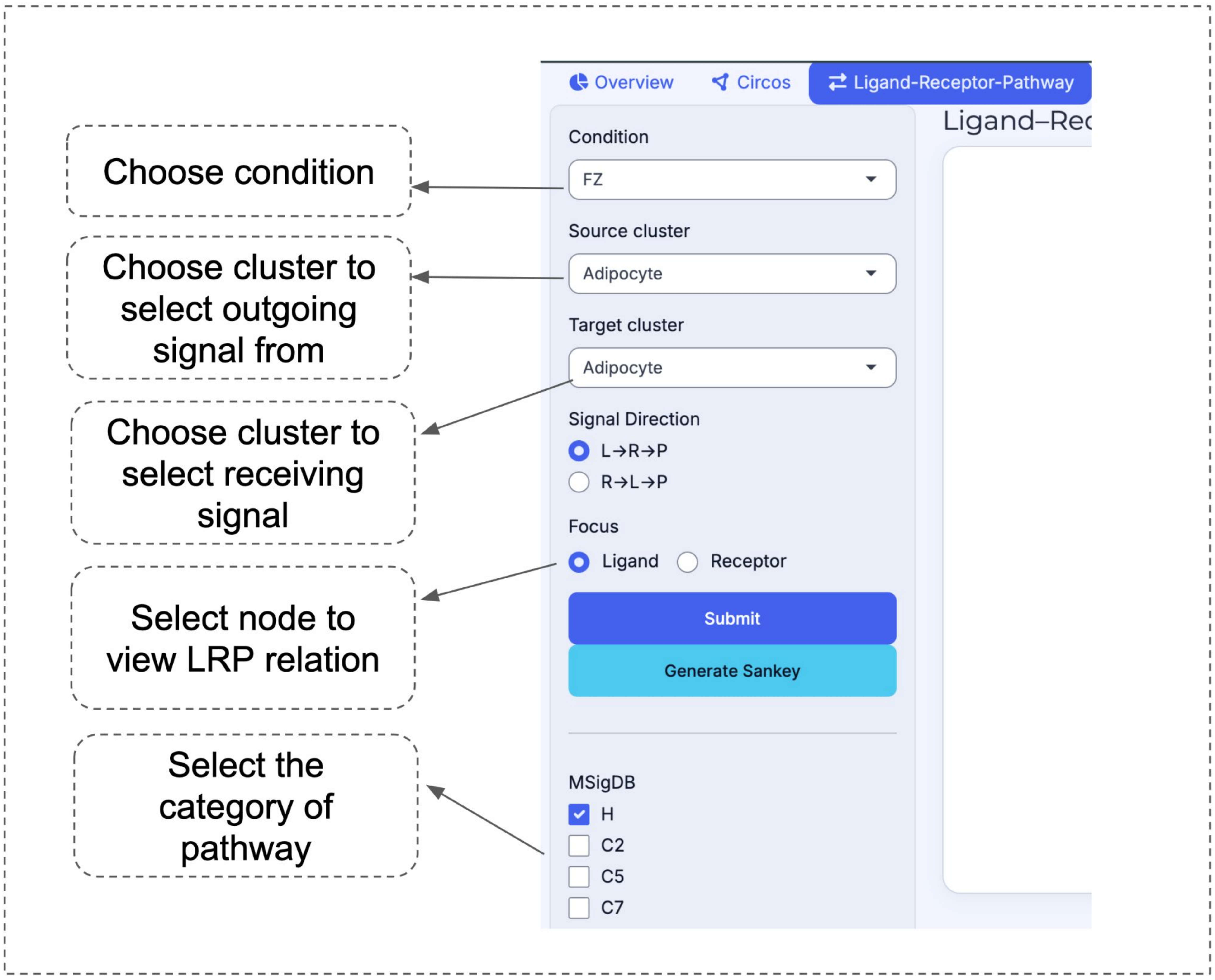

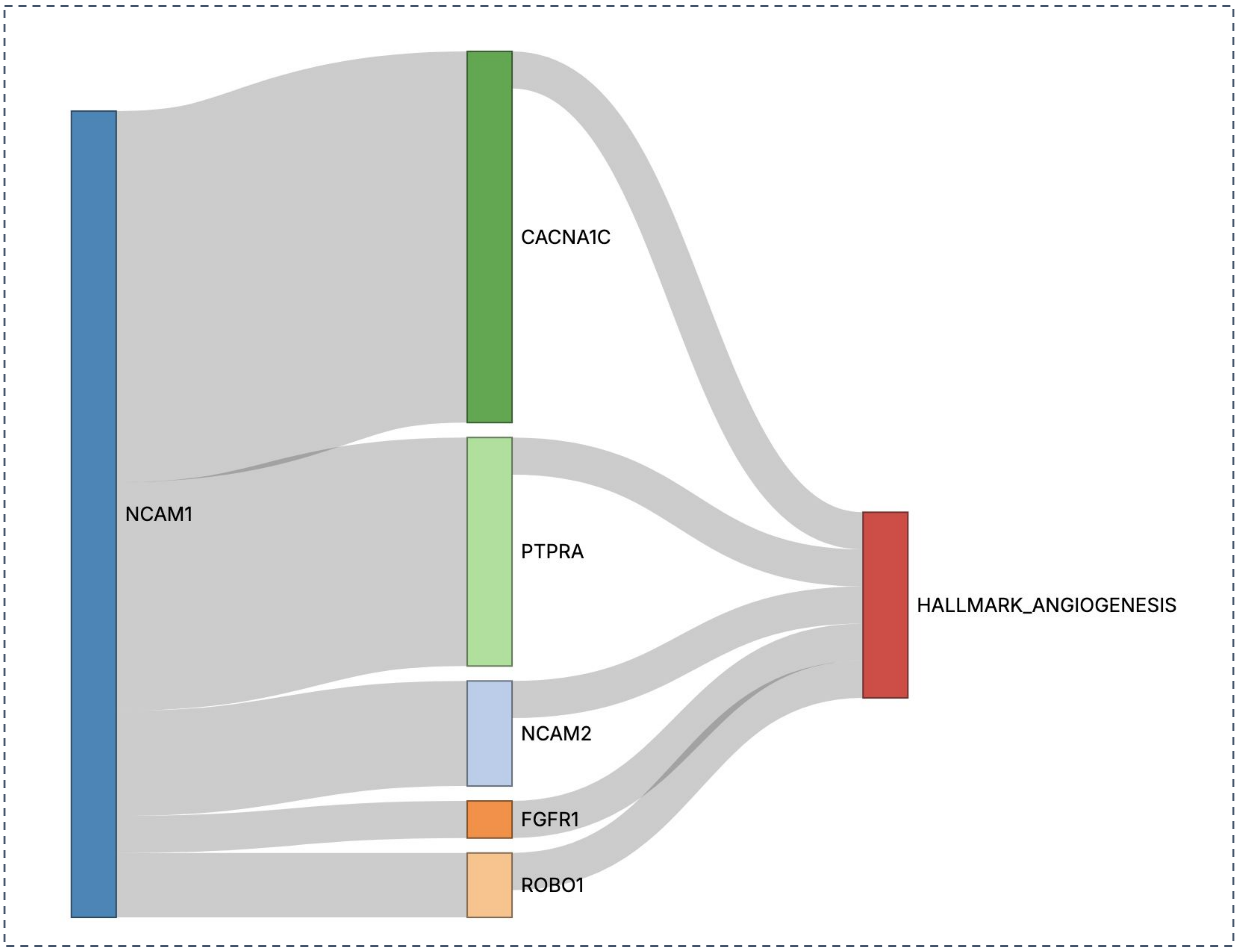

Supplementary Figure 3 :
Sankey plot showing the relation between ligand interaction with receptor, and further the pathway associated with the receptor.

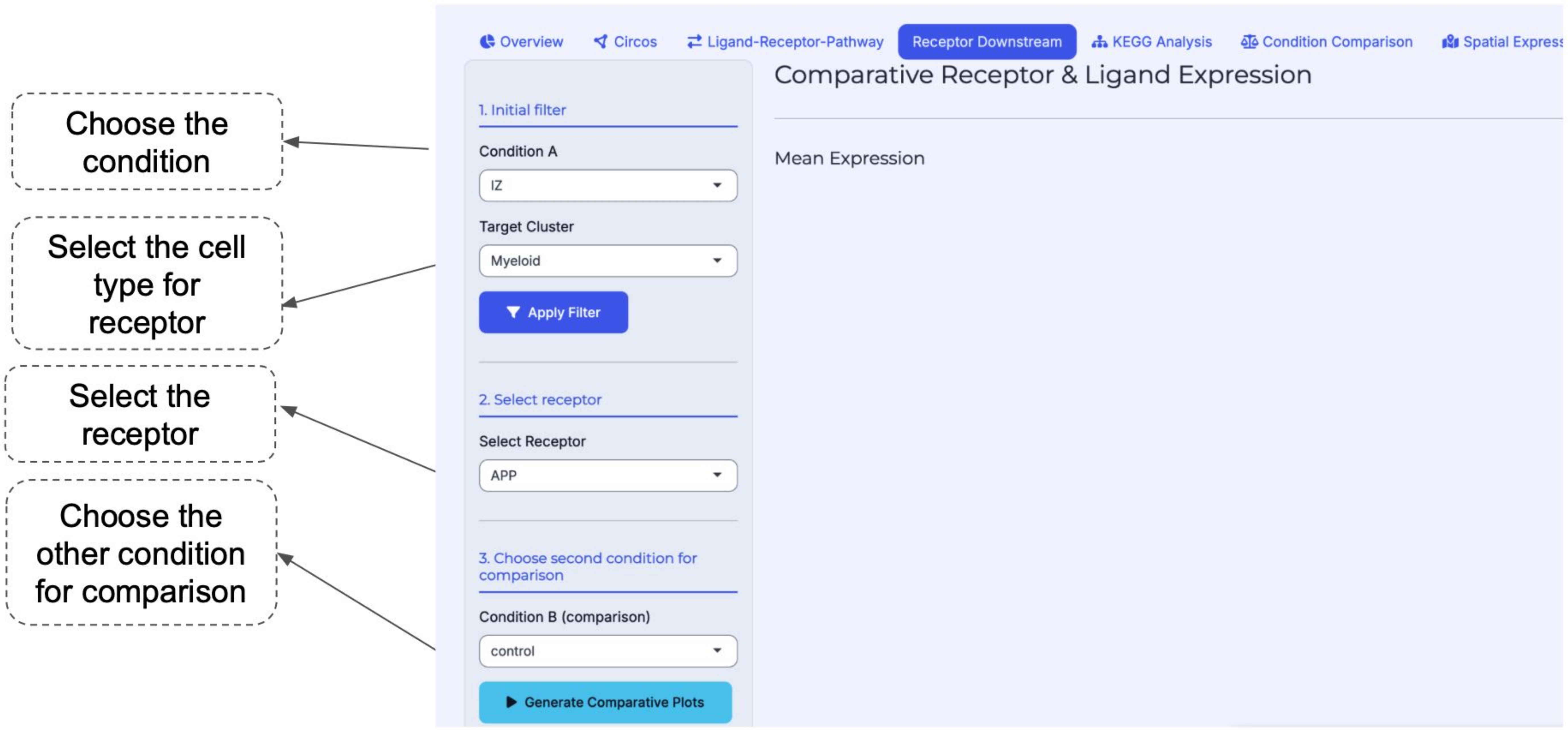

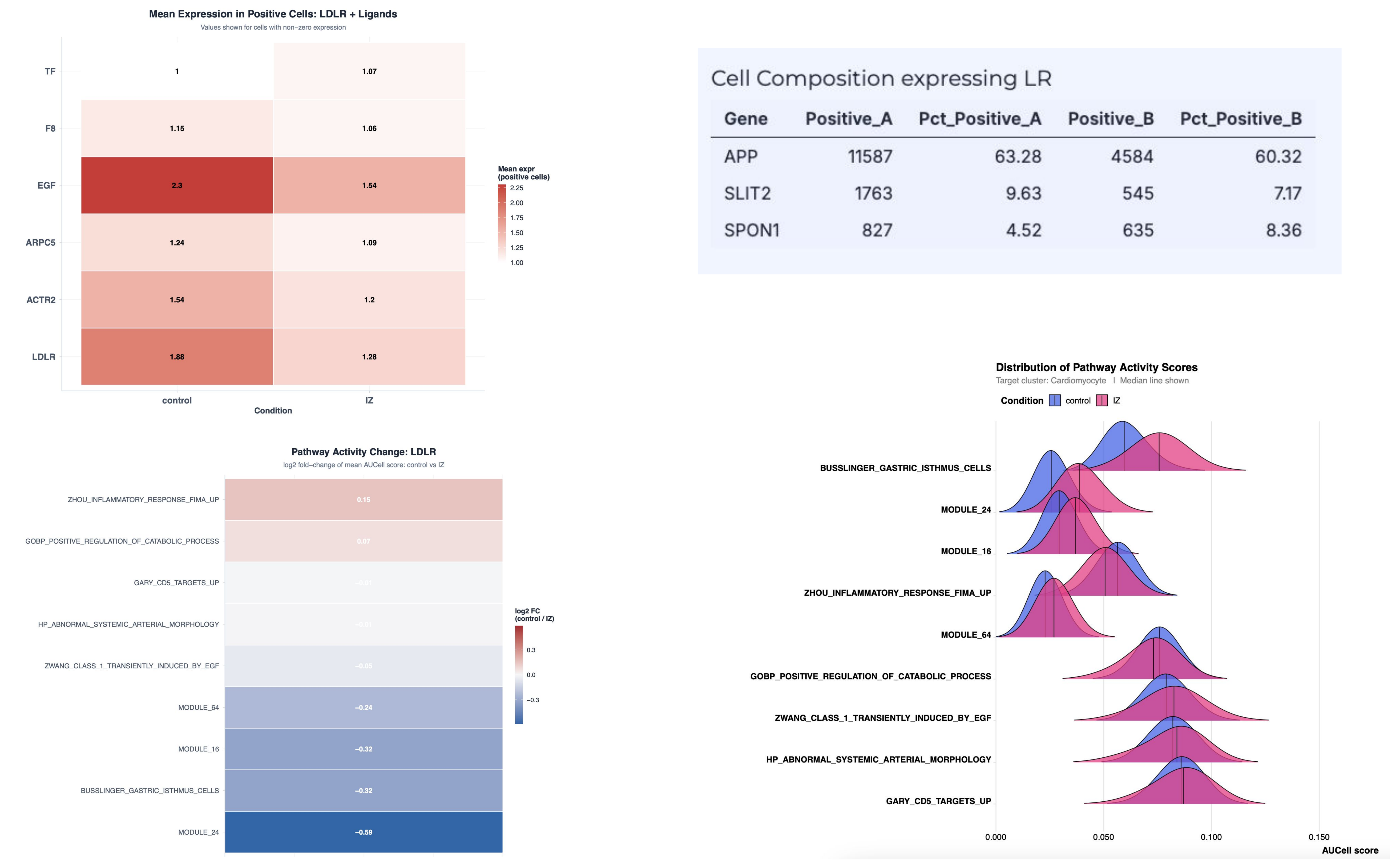

Gene	Positive_A	Pct_Positive_A	Positive_B	Pct_Positive_B
APP	11587	63.28	4584	60.32
SLIT2	1763	9.63	545	7.17
SPON1	827	4.52	635	8.36

Supplementary Figure 4::
Heatmap1 shows the expression of ligand and receptor in two conditions. Heatmap2 shows the mean AUCell score of pathway associated with the receptor in both condition. Ridge plot shows the distribution of pathway score from heatmap2 in each condition.

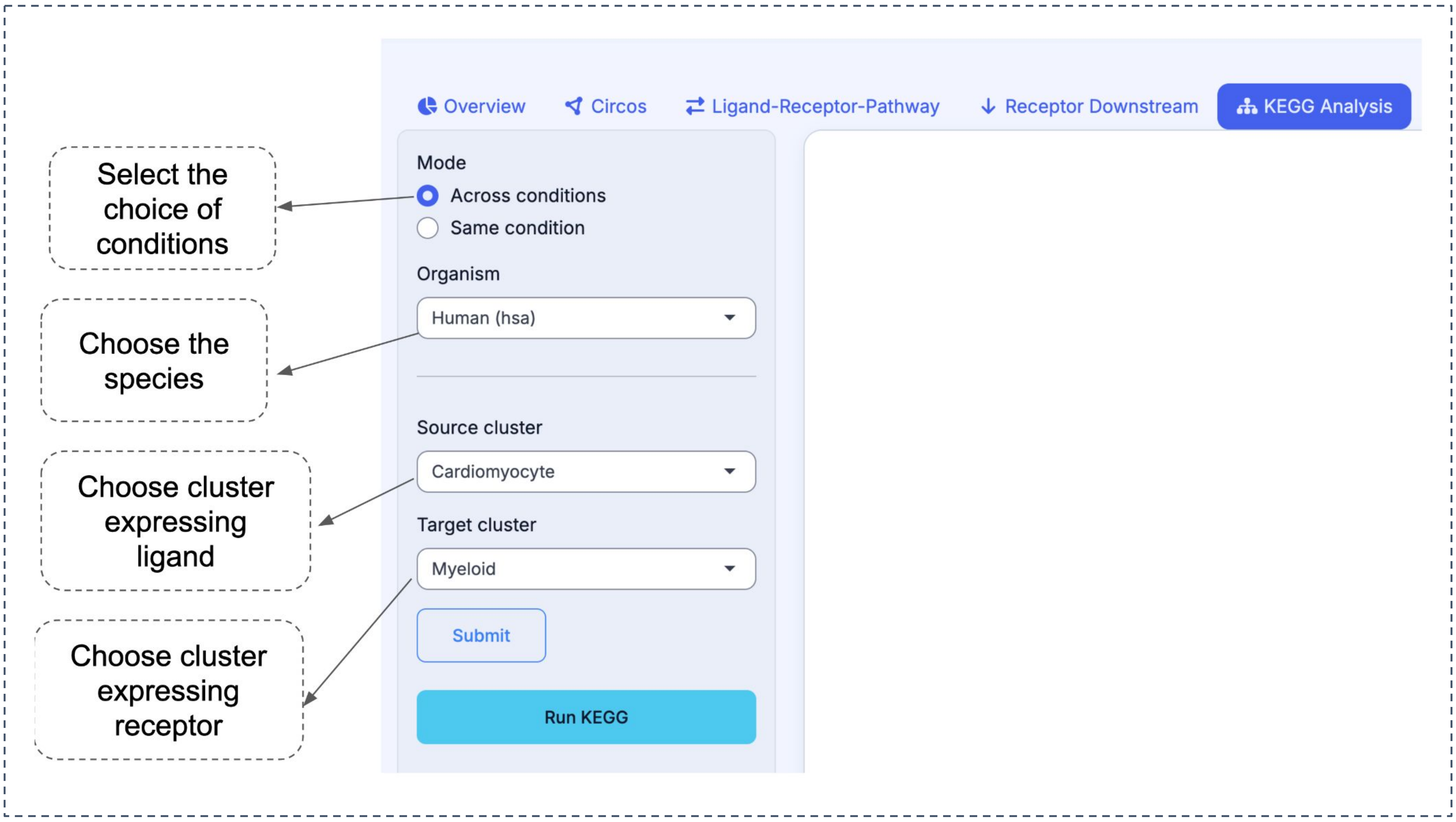

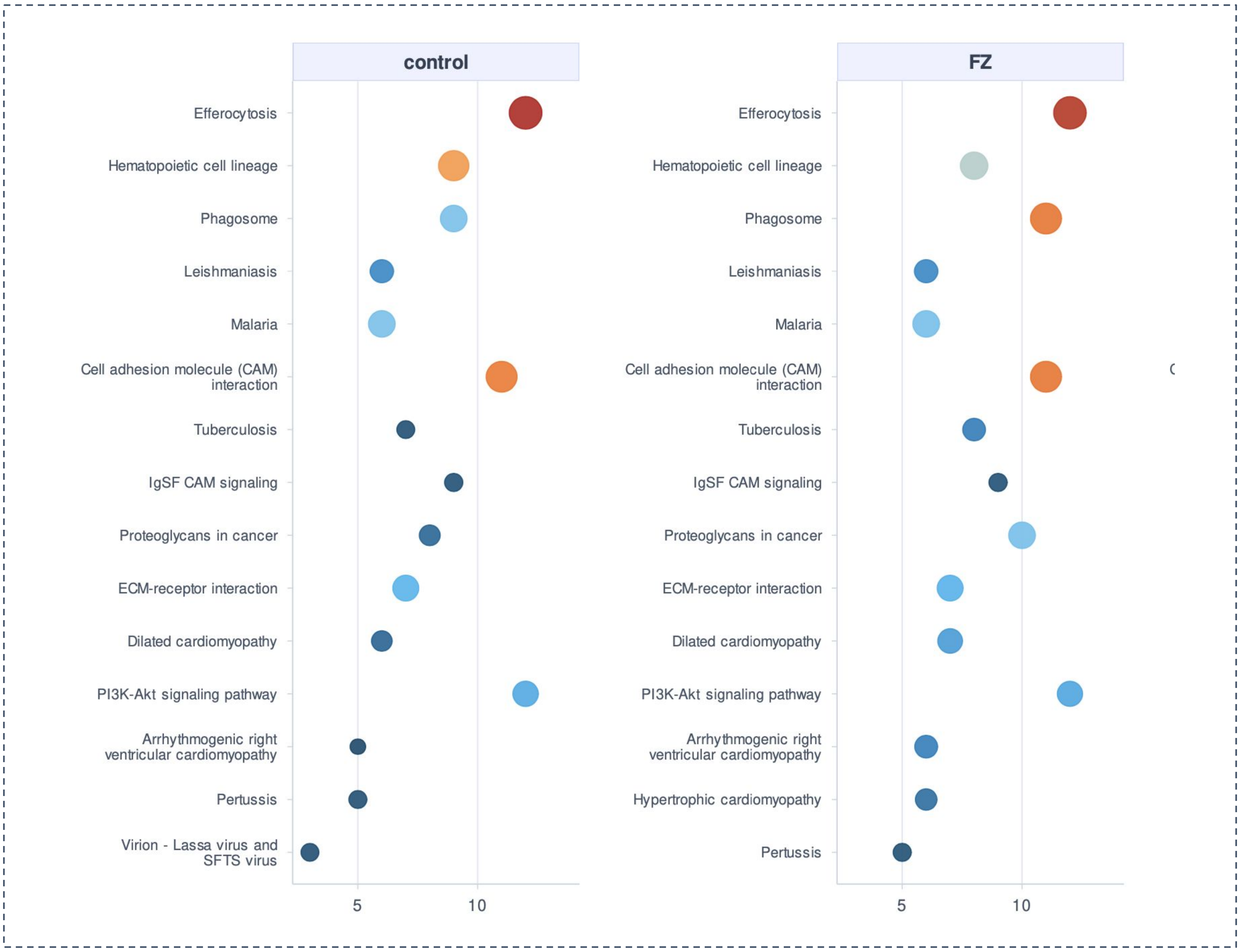

Supplementary Figure 5 :
Dot plot showing the different pathway regulation due to receptors present in chosen target - source combination for each condition.

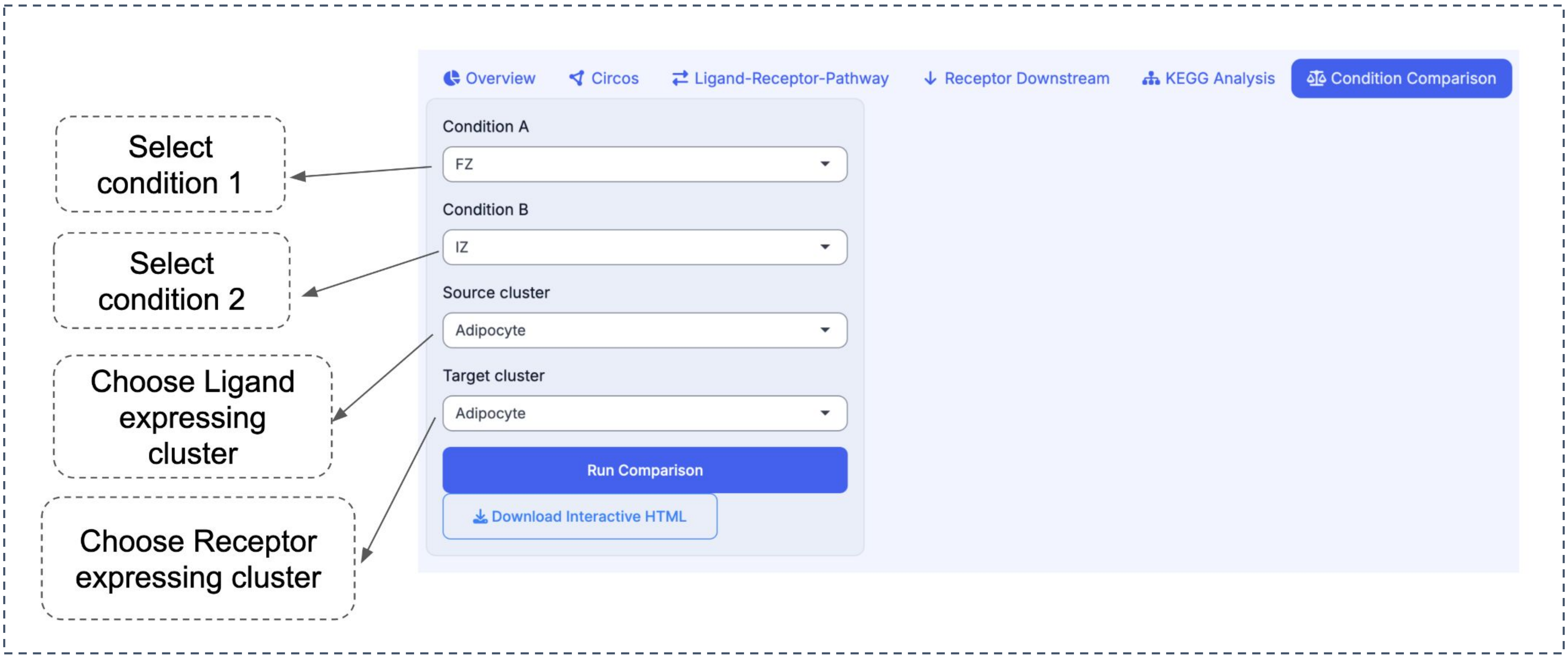

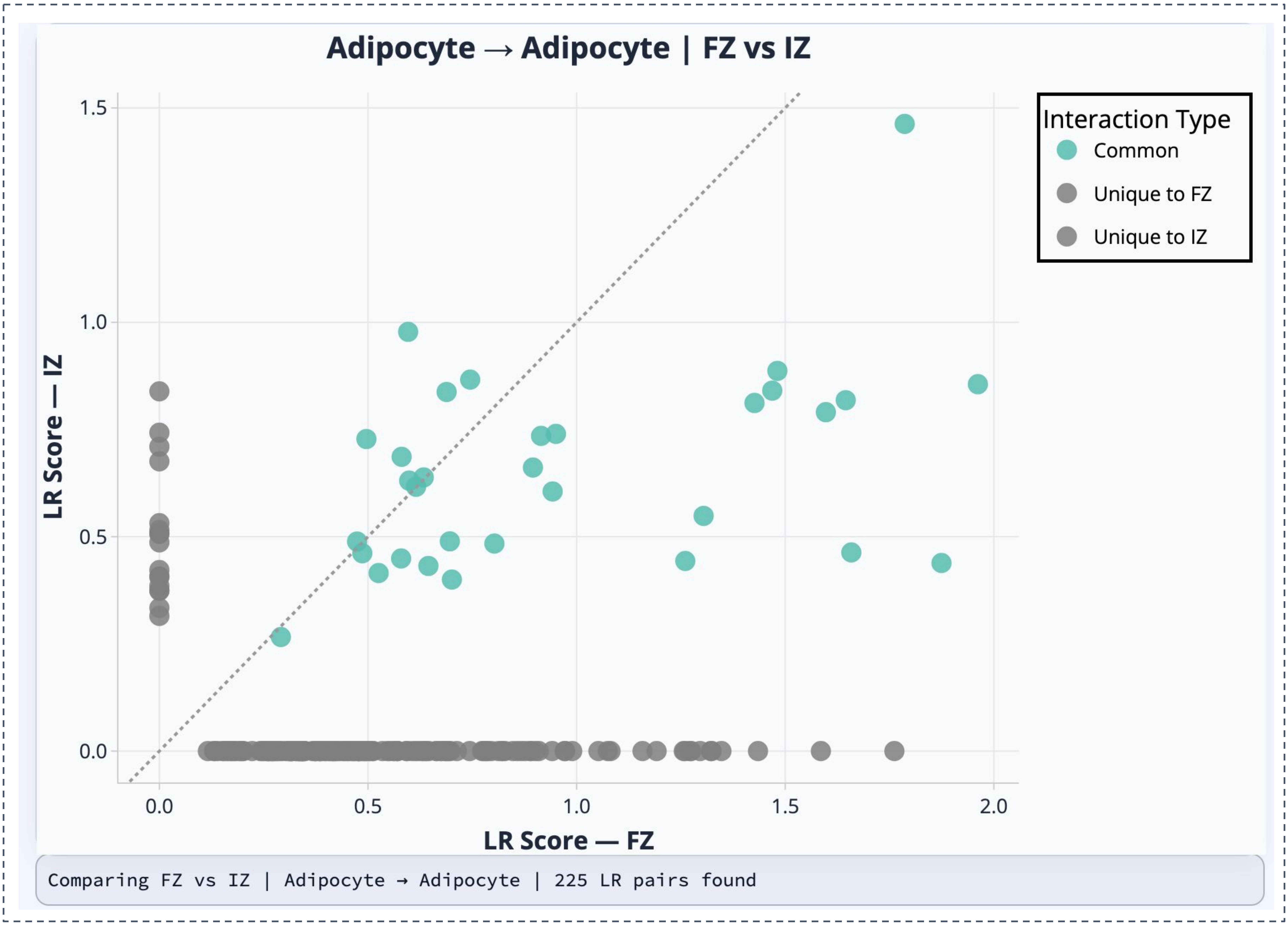

Supplementary Figure 6 :
Scatter plot depicting the unique and common ligand-receptors present in each condition. The common LR pairs are spread on the quadrant.

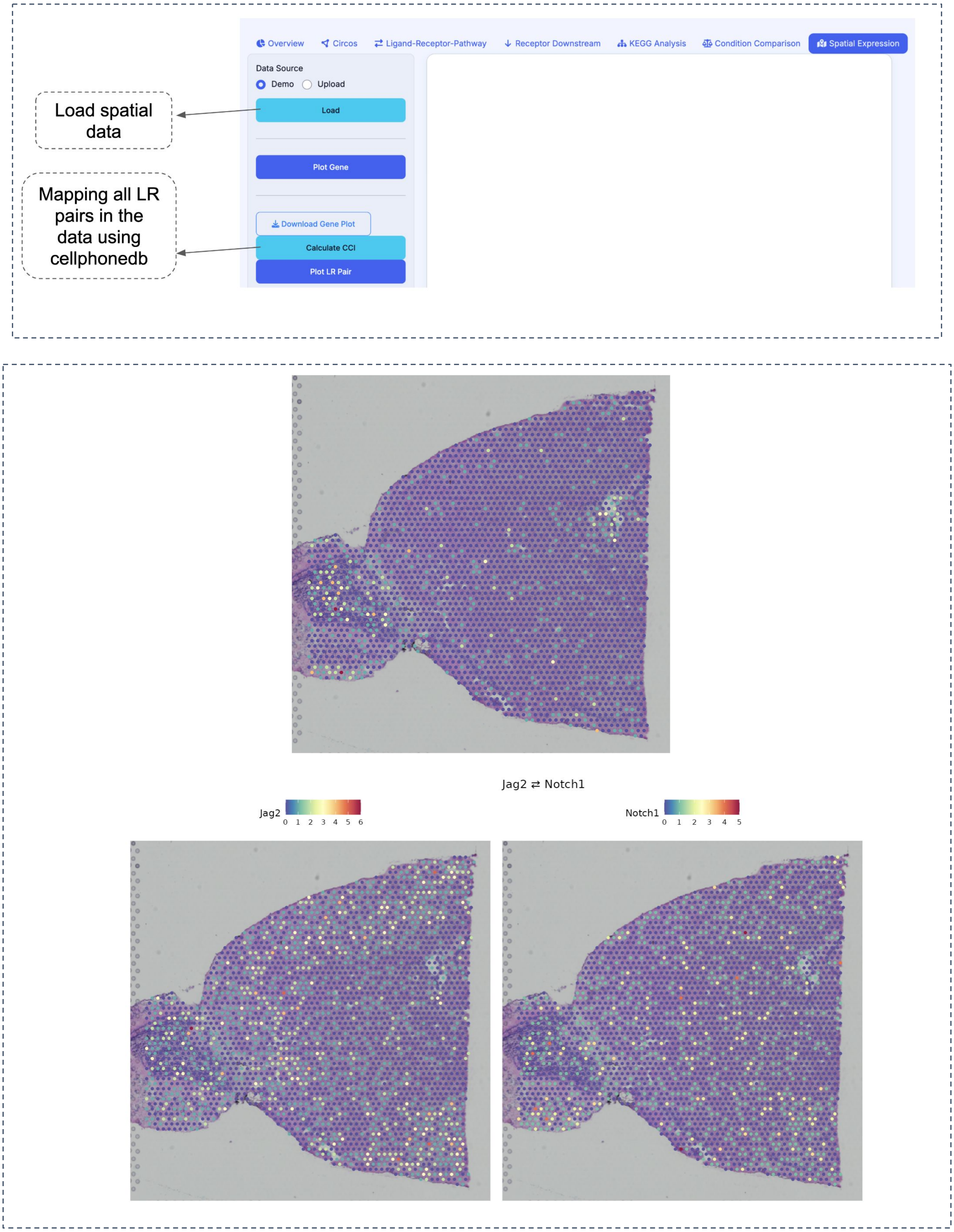

Supplementary Figure 7 :
Spatial Feature plot showing the expression of gene in space. Spatial feature plot showing the ligand-receptor pair found in cellphonedb expression in the tissue.

Supplementary Data

Supplementary Data 1: Ligand receptor pathway relationship in human MI data for control sample
The sankey graph showing the ligand-receptor-pathway relationship of selected ligands and pathways. Interactive plot showing NCAM1 ligand in mast cells in controls shows an increase in receptor interaction and and overall change in downstream pathway activity.

Supplementary Data 2: Ligand receptor pathway relationship in human MI data for samples from FZ (Fibrotic Zone)
The sankey graph showing the ligand-receptor-pathway relationship of selected ligands and pathways. Interactive plot showing NCAM1 ligand in mast cells in FZ shows an increase in receptor interaction and and overall change in downstream pathway activity.

Supplementary Data 3: Unique and common ligand-receptor pairs in condition (FZ) vs control in MI data
The interactive scatter plot shows the ligand-receptor pairs common and unique to condition. The spread of the score is shown in the quadrant, however, the unique pairs are present on the x and y axis.

Supplementary Data 4: Ligand receptor pathway relationship in human kidney KPMP data for control samples
The sankey graph showing the ligand-receptor-pathway relationship of selected ligands and pathways. Interactive plot showing EGFR receptor in mast cells in reference shows an less in ligand interaction and and overall change in downstream pathway activity.

Supplementary Data 5: Ligand receptor pathway relationship in human kidney KPMP data for (H_CKD) (Hypertension associated chronic kidney disease)
The sankey graph showing the ligand-receptor-pathway relationship of selected ligands and pathways. Interactive plot showing EGFR ligand in mast cells in (H_CKD) shows an increase in ligand interaction and and overall change in downstream pathway activity.

Supplementary Data 6: Unique and common ligand-receptor pairs in condition (H_CKD) vs control in KPMP data
The interactive scatter plot shows the ligand-receptor pairs common and unique to condition. Here, we used (H_CKD) vs reference condition for immune and PT cells. The spread of the score is shown in the quadrant, however, the unique pairs are present on the x and y axis.

Supplementary Data 7: Ligand receptor pathway relationship in human Interferon-beta1(Ifnb) stimulated PBMC data
The sankey graph showing the ligand-receptor-pathway relationship of selected ligands and pathways. Interactive plot showing TGFRB1 ligand in NK cells in stim condition and its downstream pathway activity.

Supplementary Data 8: Unique and common ligand-receptor pairs in Interferon-beta1(Ifnb) stimulation vs control in PBMC data

The interactive scatter plot shows the ligand-receptor pairs common and unique to condition. Here, we used stim vs control condition for CD14 and NK cells. The spread of the score is shown in the quadrant, however, the unique pairs are present on the x and y axis.
